# Molecular Diagnosis of Usher Syndrome: Application of Two Different Next Generation Sequencing-Based Procedures

**DOI:** 10.1371/journal.pone.0043799

**Published:** 2012-08-29

**Authors:** Danilo Licastro, Margherita Mutarelli, Ivana Peluso, Kornelia Neveling, Nienke Wieskamp, Rossella Rispoli, Diego Vozzi, Emmanouil Athanasakis, Angela D'Eustacchio, Mariateresa Pizzo, Francesca D'Amico, Carmela Ziviello, Francesca Simonelli, Antonella Fabretto, Hans Scheffer, Paolo Gasparini, Sandro Banfi, Vincenzo Nigro

**Affiliations:** 1 Cluster in Biomedicine (CBM) scrl - Genomics, Area Science Park, Basovizza, Trieste, Italy; 2 Telethon Institute of Genetics and Medicine (TIGEM), Napoli, Italy; 3 Radboud University Nijmegen Medical Center, Nijmegen, The Netherlands; 4 Institute for Maternal and Child Health - IRCCS “Burlo Garofolo”,Trieste, Italy; 5 Dipartimento di Patologia Generale, Seconda Università degli Studi di Napoli, Napoli, Italy; 6 Dipartimento di Oftalmologia, Seconda Università degli Studi di Napoli, Napoli, Italy; University of Bonn, Institut of Experimental Hematology and Transfusion Medicine, Germany

## Abstract

Usher syndrome (USH) is a clinically and genetically heterogeneous disorder characterized by visual and hearing impairments. Clinically, it is subdivided into three subclasses with nine genes identified so far. In the present study, we investigated whether the currently available Next Generation Sequencing (NGS) technologies are already suitable for molecular diagnostics of USH. We analyzed a total of 12 patients, most of which were negative for previously described mutations in known USH genes upon primer extension-based microarray genotyping. We enriched the NGS template either by whole exome capture or by Long-PCR of the known USH genes. The main NGS sequencing platforms were used: SOLiD for whole exome sequencing, Illumina (Genome Analyzer II) and Roche 454 (GS FLX) for the Long-PCR sequencing. Long-PCR targeting was more efficient with up to 94% of USH gene regions displaying an overall coverage higher than 25×, whereas whole exome sequencing yielded a similar coverage for only 50% of those regions. Overall this integrated analysis led to the identification of 11 novel sequence variations in USH genes (2 homozygous and 9 heterozygous) out of 18 detected. However, at least two cases were not genetically solved. Our result highlights the current limitations in the diagnostic use of NGS for USH patients. The limit for whole exome sequencing is linked to the need of a strong coverage and to the correct interpretation of sequence variations with a non obvious, pathogenic role, whereas the targeted approach suffers from the high genetic heterogeneity of USH that may be also caused by the presence of additional causative genes yet to be identified.

## Introduction

Usher syndrome (USH) is a group of recessively inherited disorders characterized by deafness and vision loss. Traditionally, USH is subdivided into three clinical subclasses. Visual impairment due to Retinitis Pigmentosa (RP) [1] is common to all three subtypes, which are distinguished based on the severity and progression of the hearing loss and by the presence or absence of vestibular symptoms. USH shows genetic heterogeneity: at least 11 distinct loci have been identified and 9 causative genes have been cloned. Although this classification of USH remains in clinical use, atypical clinical types have been described that defy this simple classification.

USH1 is the most severe form of USH: patients display a congenital and profound deafness associated with vestibular dysfunction as well as prepubertal onset of progressive RP [2], [3]. This form accounts for 30–40% of all USH cases [3], [4], [5], [6], [7]. To date, seven genetic loci for USH1 (*USH1B–H*) have been mapped (http://webhost.ua.ac.be/hhh/) and for five of them the corresponding genes have been identified. The genes encode: a) the actin-based motor protein myosin VIIa (*MYO7A*, *USH1B* [5], [8], [9]), whose mutations are responsible for the most common USH1 genetic subtype and accounts for approximately 30%–55% of USH1 cases [7], [10], [11], [12], [13]; b) two cadherin-related proteins, i.e., otocadherin or cadherin 23 (*CDH23*, *USH1D*) [14], [15] and protocadherin 15 (*PCDH15*,*USH1F*) [16], [17], mutated in 10–35% and 11–15% of USH1 cases, respectively [3], [4], [5], [7], [13], [18]; c) two scaffold proteins, i.e., harmonin (*USH1C*) [1], [19] that account for 6%–15% of cases [6] [13] and *SANS* (*USH1G*) [20] responsible for about 7% of cases [6].

The USH2 type is less severe and is characterized by moderate to severe congenital deafness, with a high-frequency sloping configuration. Owing to the overlap between types I and II in clinical appearance and age of onset, visual symptoms are not considered reliable predictors of USH type in individual cases [2], [3], [21]. Three genetic loci have been reported so far in USH2 (*USH2A*, *USH2C* and *USH2D*) and the corresponding genes have been identified. Mutations in the *USH2A* gene, encoding usherin, underlie the most common form of USH2 accounting for up to 85–86% of cases [7], [13], [22], [23]. Mutations in the *USH2C* and *USH2D* genes are much rarer [7], [13], [24], [25]. The protein encoded by the *GPR98* gene at the *USH2C* locus is a member of the serpentine G-protein coupled receptor superfamily [24]. Defects in the *DFNB31* gene, a PDZ (post-synaptic density, disc-large, Zo-1 protein domains) domain-containing scaffold protein, are responsible for *USH2D* and nonsyndromic hearing loss (*DFNB31*) [25], [26]. Finally, USH3 is characterized by variable onset of progressive hearing loss, variable onset of RP, and variable impairment of vestibular function and is caused by mutations in the *USH3A* (clarin-1) gene, located on 3q21-q25 [27], [28]. USH3 is the less common form of Usher syndrome with a prevalence of 2–4% within all USH cases [6], [7], [13].

Identification of the USH causative mutations is important for early diagnosis, genetic counseling and prenatal diagnosis. Despite the fact that most Usher syndrome patients can be reliably grouped into one of the three main clinical classes, a comprehensive molecular diagnostics protocol for Usher syndrome has been hampered by genetic heterogeneity, the large number of exons to analyze and by the high costs associated with application of conventional techniques [29]. Current diagnostic strategies for Usher syndrome include: a) the use of genotyping microarrays based on the arrayed primer extension (APEX) method that can detect the presence of previously reported mutations and has proved to be an adaptable and affordable mutation screening tool [29], [30], [31]; and b) complete exon sequencing of all known Usher syndrome genes by Sanger sequencing, which was recently demonstrated to significantly improve diagnostic efficiency for this condition [10], [13], but it is a demanding procedure in terms of both cost and time.

Newly developed molecular technologies are therefore needed to facilitate the discovery of underlying gene mutation early in life and to provide estimation of its prevalence in at risk pediatric populations thus laying a foundation for its incorporation as an adjunct to newborn hearing screening programs. During the past five years, new high-throughput DNA sequencing technologies collectively referred to as next generation sequencing (NGS) have emerged [32], [33], [34]. NGS is nowadays widely used in biomedical research [35], [36], [37], [38], [39], but its applications in molecular diagnostics are still limited [40], [41], [42], [43]. NGS allows to efficiently sequence an entire genome, an exome, or specific genomic regions. The latter can be achieved by using one of several enrichment methods including long-range PCR (LR-PCR), fragment-capture using solid surface arrays and in-solution oligonucleotide capture [44], [45], [46], [47]. In this pilot study, we report the results obtained by using both exome and targeted sequencing on human genomic DNA samples from Usher Syndrome patients.

## Results

**Figure 1:**
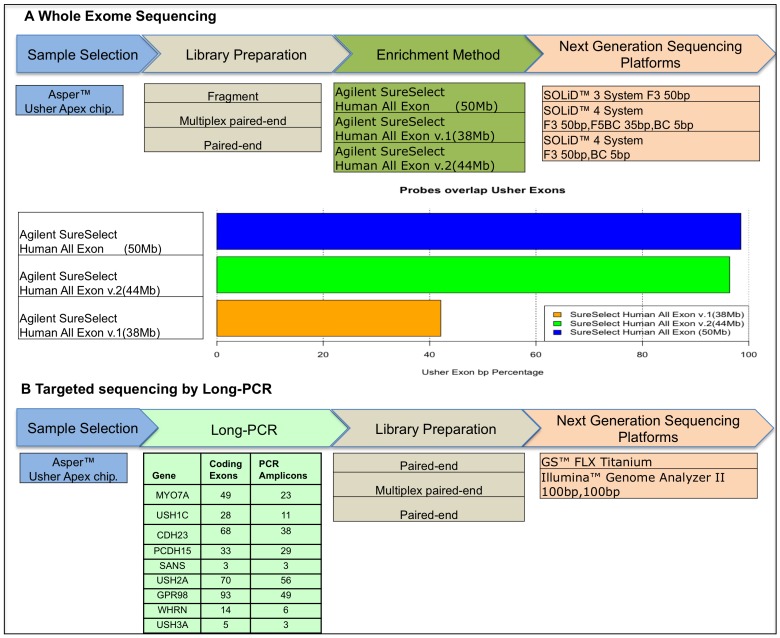
Workflow of the next generation sequencing strategies used. A) whole exome sequencing workflow. Samples have been pre-screened using an Apex-based Usher genotyping microarray; library preparations prior to enrichment include fragment single reads or Paired-End preparation. Three different types of enrichment methods have been used; each enrichment probe sets overlap at different extent to the RefSeq coding regions of Usher genes (horizontal bars). Sequencing protocols include single 50 bp reads on the Solid3 System, single 50 bp read on Solid4 System, Paired-end reads 50 bp+35 bp on Solid4 System. B) Long-PCR sequencing workflow. Samples have been pre-screened using Usher Apex microarray, Long-PCR approach produced 218 PCR amplicons used as input for the for *Fragment* and *Paired-End* library preparation. Sequencing was performed using both GS-FLX and GAII Systems.

To evaluate whether the currently available NGS technologies are ready for USH diagnostics we used: a) Whole exome sequencing with analysis restricted to known USH genes and b) targeted resequencing of the known USH genes starting from long-range PCR products. A schematic overview of the strategy is shown in Figure 1.

### Whole Exome sequencing

**Figure 2:**
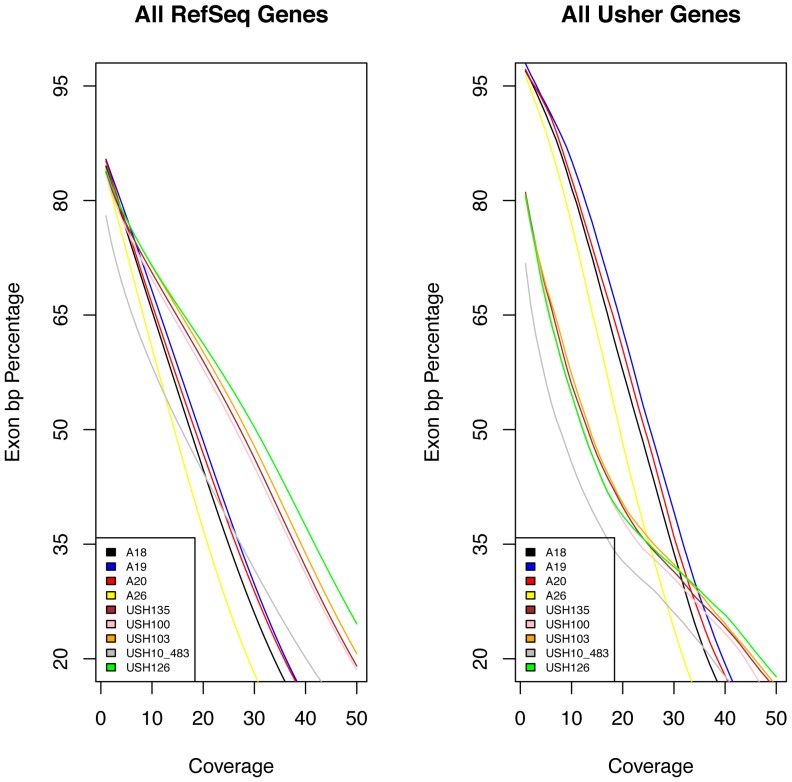
Coverage data for whole exome sequencing. A) Relationship between the minimum depth coverage and the extent of basepairs of RefSeq exons sequenced (shown in percentage). B) Relationship between the minimum depth coverage and the extent of basepairs of Usher exons sequenced. Solid colored lines represent different samples, x axis: minimum coverage increasing from left to right up to 50×; y axis: percentage of exons sequenced.

We tested nine different Usher patient samples, which previously turned out to be negative for known mutations in Usher genes as assessed by APEX microarray screening [31]. In the analysis, we focused only on the coding exons and the related splice junction sites of known Usher genes. Each obtained sequence was analyzed according to a specific workflow as for the different protocols and platforms used (see Information S1 and Figures S1 and S2). An average of 12 Gb of sequence was generated per affected individual (Figure S3). A total of 43.3 million non-duplicated reads could be mapped to the genome. We found high correlation values (r^s^>0.9) among coverage depth positions of samples processed using the same enrichment method. We decided to consider the minimum and not the mean of obtained coverage depth to better evaluate the detection of variations in any coding position. The library and sequencing chemistry used influenced the overall results in terms of sequence obtained: on average 58 million reads were obtained for samples sequenced with SOLiD4 Paired-end protocol (Figure 2, samples labeled with the “USH” prefix) while 30 million reads were obtained for SOLiD3 fragment sequencing (Figure 2, samples labeled with the “A” prefix). The sequence coverage of the USH genes was comparable with all the Human RefSeq genes regarding the increase of minimum depth demand (Figure 2A–B). Within the Usher genomic regions we identified approximately 100 total sequence variants per sample with an average coverage of 20×. In the process of sequence variant calling, a) we selected those variants that were located in translated regions; b) we selected those variants supported by reads on both DNA strands in human genome; c) we removed those variants deposited in the dbSNP database (version 135), 1000 human genome dataset, and in our in-house exome database that includes healthy individuals and patients with non-ocular conditions. A schematic workflow of our filtering step procedure and data analysis, similar to the scheme reported by Wei, X. et al [48] is depicted in Figure S4. This filtering process narrowed down the observed variations to 1–2 per samples (Table 1 and Table S3), which were all confirmed by Sanger Sequencing. An overall number of 12 variations in 5 known USH genes was detected. The variation effect was predicted following a multi-step analysis available on the Usher Syndrome Missense Analysis (USMA) website (https://194.167.35.160/cgi-bin/USMA/USMA.fcgi). Three variants were already reported in USHbase database as neutral (*MYO7A*: c.4697C>T), probably neutral (*USH2A*: c.14074G>A) and likely pathogenic (*USH2A*: c.3176C>T) while the remaining 9 were not described so far (Table 1). *In silico* analysis of the variations allowed us to infer the pathogenic role for 3 out of 9 variants, thanks to the availability of secondary and 3D structure analysis of the corresponding protein regions (Table S1).

**Table 1:** Sequence variants of USH patients identified by whole exome sequencing.

USH Sample (Type)	Gene	Nucleotide change	Amino Acid change	Enrichment	Zygosity	Classification(1)	Apex
**A18 (1)**	***MYO7A***	c.4411T>C	p.S1471P	Human all Exon (50 Mb)	Hetero	Unreported	N.A.
	***CLRN1***	c.218A>G	p.Q73R	Human all Exon (50 Mb)	Hetero	Unreported	N.A.
**A19 (1)**	***MYO7A***	c.4697C>T	p.T1566M	Human all Exon (50 Mb)	Hetero	Neutral	N.A.
	***GPR98***	c.10577T>C	p.M3526T	Human all Exon (50 Mb)	Hetero	Unreported	N.A.
**A20 (2)**	***USH2A***	c.4663G>T	p.G1555C	Human all Exon (50 Mb)	Hetero	Unreported	N.A.
	***USH2A***	c.14219C>A	p.A4740D	Human all Exon (50 Mb)	Hetero	Unreported	N.A.
**A26**	***-***	-	-	Human all Exon (50 Mb)	-	-	-
**USH100 (2)**	***USH2A***	c.14074G>A	p.G4692R	Human all Exon v2(44 Mb)	Hetero	UV1 (probably neutral)	N.A.
	***USH2A***	c.9203delT	p.V3068fs	Human all Exon v2(44 Mb)	Hetero	Unreported	N.A.
**USH103 (2** (2) **)**	***CLRN1***	c.619C>T	p.R207*	Human all Exon v2(44 Mb)	Homo	Unreported	N.A.
**USH126 (2)**	***PCDH15***	c.4880T>C	p.V1627A	Human all Exon v2(44 Mb)	Homo	Unreported	N.A.
**USH135**	***-***	-	-	Human all Exon v2(44 Mb)	-	-	-
**USH10_483 (2)**	***USH2A***	c.3176C>T	p.P1059L	Human all Exon v1(38 Mb)	Hetero	UV3 (likely pathogenic)	p.P1059L
	***GPR98***	c.11974G>A	p.D3992N	Human all Exon v1(38 Mb)	Hetero	Unreported	N.A.

(1) We annotated the resulting variation according the USHbase database (https://grenada.lumc.nl/LOVD2/Usher_montpellier/USHbases.html) and the 9 variants not present in the database have been classified as unreported.

(2) Clinical diagnosis compatible with USH type 3.

Notably, only for three samples (A20, USH126, USH103) we were able to identify two putatively pathogenic sequence variations in the same gene while for two samples (A26, USH135) we were not able to identify any putative mutation.

### Targeted Resequencing

**Figure 3:**
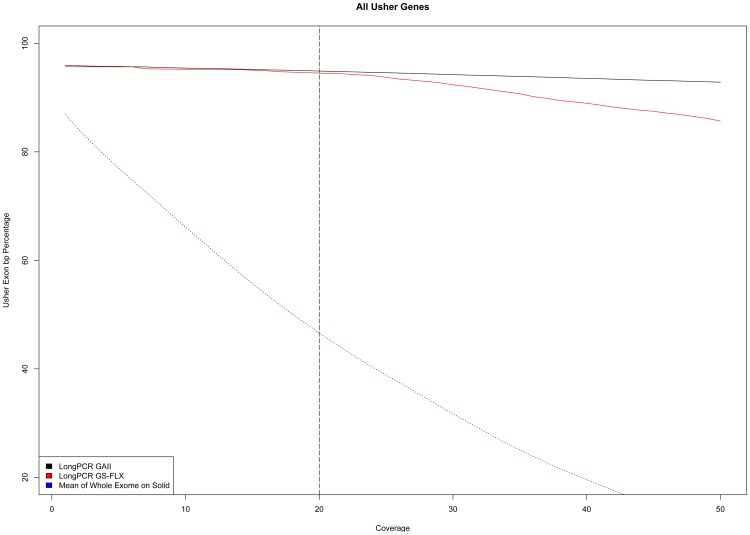
Coverage data for long-PCR NGS sequencing. Relationship between the minimum depth coverage and the extent of basepairs of Usher exons sequenced. Solid colored lines represent sample sequenced on different platforms, whereas the dotted line is the average representation obtained from the nine sample of Figure 1. x axis indicates the minimum coverage increasing from left to right up to 50× while the y axis indicates the percentage of Usher exon basepairs sequenced.

As an alternative strategy for the molecular analysis of Usher genes, we tested the efficacy of targeted resequencing. We designed an in-house enrichment protocol based on 218 Long-PCR fragments (Table S2) covering all the known exons of the 9 Usher genes. We carried out this analysis on a set of three Usher patients different from those analyzed by exome sequencing. All the analyzed patients had been prescreened using the APEX-based genotyping chip and some putative mutations had already been identified (Table 2). The obtained Long-PCR amplicons were equimolarly mixed and subsequently split in two parts to use the same starting material for the different library preparations according to the NGS platform (see Material and Methods). We obtained 5 Gb of sequences using the Illumina GAII and 45 Mb using the GS-FLX platform corresponding to 14.3 millions and 0.22 millions of non-duplicated mappable reads, respectively. Overall, as expected, this Long-PCR approach yielded a much higher coverage in Usher genes as compared to whole exome sequencing and about 94% of the Usher gene exons show a mean coverage higher than 20× (Figure 3). Since the same long-range PCR library was analyzed using two different sequencing platforms, we could also compare the two procedures. Considering the entire Long-PCR product as target, we can clearly distinguish a better on-target efficiency of the Illumina GAII Paired-End Libraries (99% On-Target) compared to GS-FLX (90% On-Target Figure S5). This difference seems to be increased using the open source BWA mapping results while platform proprietary mapping software show a lower Off-Target for the same GS-FLX data (95% On-Target). Among Long-PCR products, we registered a similar coverage performance of GS-FLX up to 24–25×, but above that value the curves show a more severe drop for GS-FLX compared to GAII (Figure 3). We decided to further investigate the trend by checking for systematic biases in the coverage among our Long-PCR results. By investigating the coverage distribution of each single amplicon, we observed an uneven distribution of the coverage, which could not be ascribed to a random effect. Using the data from different samples we uncovered a position-dependent coverage correlation with a Spearman Rank Order Correlation coefficient of 0.82 *r^s^* (Figure S6).

**Table 2:** Sequence variants of USH patients identified by Long-PCR sequencing.

USH Sample (Type)	Gene	Nucleotide change	Amino Acid change	Enrichment	Zygosity	Classification(1)	Apex
**Sample1 (2)**	***CDH23***	c.1423G>C	p.V475M	Long-PCR	Hetero	N.A.	N.A.
	***USH2A***	c.2137G>C	p.G713R	Long-PCR	Hetero	UV2 (likely neutral)	p.G713R
**Sample2 (2)**	***USH2A***	c.2229delG	p.E767fs	Long-PCR	Hetero	Pathogenic	p.E767fs
	***CDH23***	c.3625A>G	p.T1209A	Long-PCR	Hetero	UV2 (likely neutral)	p.T1209A
**Sample3 (1)**	***MYO7A***	c.77C>A	p.A26E	Long-PCR	Hetero	Pathogenic	p.A26E
	***MYO7A***	c.3827C>A	p.S1276*	Long-PCR	Hetero	Unreported	N.A.

1 Classification based on the USHbase database https://grenada.lumc.nl/LOVD2/Usher_montpellier/USHbases.html.

For sequence variant calling, we used the same criteria described for the analysis of exome-enriched samples. On average, we obtained a total of 129 sequence variants per sample that narrowed down to a few variations with our selection criteria. In addition to the expected mutations, i.e., those identified by microarray analysis, two unreported mutations were identified (Table 2). Sample 1 presents an unreported heterozygous missense mutation in *CDH23*: c.1423G>C;p.V475M, classified as probably-damaging according to Poliphen2 prediction and located in a domain where other pathogenic variants have previously been reported [49]. In the case of Sample 3 we identified an unreported *MYO7A* variation (*MYO7A*: c.3827C>A;p.S1276*) that complements with the previously reported variation (*MYO7A*: c.77C>A;p.A26E) identified through APEX screening. Interestingly many conservation scores (Placental Mammal Basewise Conservation,Vertebrate Basewise Conservation, Primate Basewise Conservation) drastically decrease exactly after the above nonsense mutation (Figure S7).

## Discussion

Molecular diagnosis in Usher syndrome is hindered by significant genetic heterogeneity, the large size of some of the Usher genes, and the high number of polymorphic variations in genes such as *MYO7A* and *USH2A*. Furthermore, a digenic inheritance has been proposed in some cases of Usher syndrome [10], although not yet universally accepted [13], which further complicates the elucidation of the molecular basis of genotype-phenotype correlation. Therefore, the molecular analysis of known genes using Sanger sequencing is challenging and cannot be offered routinely [10], [13]. The goal of our study was to test the promising NGS technologies that could be applied to the molecular diagnosis of Usher syndrome.

To test all the available options for diagnostic NGS, we investigated to what extent the current NGS technologies can be used in this process and we compared the efficacy of whole exome approaches *vs.* gene-specific approach based on Long–PCR enrichment. In addition, we obtained sequence data with all different NGS platforms. To our knowledge, this is the first attempt to use NGS protocols in the molecular diagnosis of Usher syndrome.

The first evidence we gathered is that when using whole exome sequencing the percentage of Usher genes represented ranged from a maximum of 98% to a minimum of 76% at low sequence coverage (Figure 2B and Figure S2). However, those values rapidly decrease as the coverage demand increases. Without a substantial increase of the number of the sequencing reads and hence of the sequencing costs/sample, the whole exome approach can be insufficient for USH diagnosis. For example, setting a minimum coverage of 30×, only 50% of the USH sequences are available for analysis. In addition, it is also evident that the enrichment protocol produces an uneven coverage with peaks and falls even in closely adjacent genomic regions depending on the enrichment protocol used (see Information S1 and Figures S8 and S9), as shown by high correlation values between samples processed using the same enrichment protocol. Even if newer versions of enrichment kits usually work better than previous ones, when looking at single genes it is possible to find some specific exceptions (like in the case of *USH2A*) in which the earlier version of the kit gives a better coverage curve (Figure S3).

In contrast, the Long-PCR-based enrichment leads to an almost constant curve guaranteeing a higher minimum depth coverage. Our high correlation value, between samples processed for Long-PCR, seems to indicate a systematic non uniform distribution in our approach. This may be due to bias in the PCR products since no valid correlation with GC composition or length of the sequenced region can be addressed. Nevertheless, the comparison of the exon coverage between the Long-PCR and in-solution method shows that the Long-PCR approach guarantees always a higher number of exons sequenced for any selected coverage (Figure 3). This result encourages the development of protocols to obtain DNA templates prepared by multiplex Long-PCR in order to decrease costs and workload/sample.

We adopted a high stringency multi-steps filter criteria to select the pathogenic variants. This approach lowered the number of false positive variants, although it could raise the number of false negatives leading to an underestimation of the real number of variants. The high number of unreported variants (approximately 1 variant per sample) shows that, in the process of designing any strategy for USH molecular diagnosis, the high prevalence of novel mutations is a major issue, as already suggested in some recent publications [10], [13]. Sequencing of all known USH exons and not only the screening of known mutations is required for proper molecular diagnosis and an accurate genetic counseling. In this light, we should pay attention to the lack of an adequate coverage due to the enrichment step, an issue that will be solved with the technical improvements of the capturing methods.

We observed that NGS of long-PCR products did improve the detection rate of mutations over APEX screening, but not over the more demanding Sanger methods [10]. This suggests that the genetic heterogeneity of USH is much higher and makes the NGS technologies cost-effective in terms of diagnostic power only in the cases due to mutations in the known genes. Although NGS can be envisaged to have multiple applications in clinical diagnostics, the technology is currently complex and requires attention to technical issues, including sequencing template enrichment and management of massive data. In particular, for lower complexity samples a point of diminishing returns is reached when the number of counts per sequence results in oversampling with no increase in data quality.

Out of the twelve USH patients analyzed, we could genetically solve five of them as we identified two presumably pathogenic variants in the same gene (A20, USH100, USH103, USH126, and Sample3). We identified a single heterozygous putative pathogenic variant in five other patients, while we could not recognize any pathogenic sequence variations in the remaining two USH patients (A26 and USH135, Table 1). Our results are overall in agreement with those on larger cohorts of USH patients by Sanger sequencing [10], [13]. The higher percentage of genetically unresolved cases (2/12 corresponding to 16% of patients analyzed) with respect to previous studies can be explained by the fact that for the whole exome sequencing analysis we selected USH patients that were not found to harbor any known mutation in USH genes by APEX screening. We thus enriched our starting dataset for patients with higher probability to carry mutations in novel, yet to be identified USH genes.

In conclusion, the constant decrease in costs of NGS procedures will make them even more attractive in the near future. Even if nowadays whole genome approaches do not seem to fulfill the requirements of a complete molecular screening of Usher Syndrome, they nevertheless remain a potential precious tool for the identification of new players in disease.

## Materials and Methods

### Patient selection

The patients examined in this study underwent a screening visit at the diagnostic centers of either Trieste or Naples that included a basic ophthalmic consultation, central visual acuity (CVA), Goldman visual field (GVF), fundus oculi, standard electroretinography (ERG), and a series of periodic control visits. The patients also provided a detailed medical history and underwent genetic counseling to identify hereditary patterns. Each patient underwent a complete ear, nose and throat (ENT) and audio-vestibular examination, including otomicroscopic examination, audiometry, and an auditory brainstem response (ABR) for threshold assessment of patients less than 5 years of age. Blood samples were collected and genomic DNA was extracted from blood samples using standard techniques. All patients studied entered the diagnostic centers of Trieste or Naples and signed an appropriate consent form for genetic testing as well as forms related to privacy of data. Approval for the study was obtained by the Second University of Naples and by IRCCS-Burlo Garofolo of Trieste Ethics Committees.

### Exome Enrichment

For Agilent exome enrichment 3 µg genomic DNA was used. We used ABI SOLiD optimized kits (Agilent, Santa Clara, CA, USA), following the manufacturer's instructions. The different enrichment kits are reported in Information S1. Briefly, for every 3 µg DNA, we diluted the genomic DNA and, using a Covaris station, sheered the genomic DNA to 150 base pair fragments. The purified obtained samples were end repaired, adaptor ligated and the obtained library amplified according to the SureSelect Target Enrichment protocol. The final step of hybrid capture selection provided an enriched library that has been quality assessed with Agilent 2100 Bioanalyzer. The enriched exome libraries were subsequently used for e-PCRs following manufacturer's instructions (Life Technologies, Carlsbad, CA, USA), based on a library concentration of 0.5 pM. DNA sequencing was performed with the use of the SOLiD system that involves ligation-based sequencing and a two-base encoding method in which four fluorescent dyes are used to tag various combinations of dinucleotide, reducing the risk of false positive determinations. Differences in the sequencing chemistry used are reported in Information S1.

### Long-PCR Design

Primers were chosen using the web-based program Primer3 (PRIMER3, primer3_www.cgi v 0.2). Each oligo contained 26–32 nt with a predicted melting temperature higher than 59°C and lower than 66°C with a GC content lower than 40% where not possible (14 primers) we selected primers with higher GC contents. Primers were also checked by BLASTn against the NCBI data bank genome for specificity (http://www.ncbi.nlm.nih.gov/BLAST). For long-PCR, 100 ng of genomic DNA was used for each PCR. We used Takara LA buffer or equivalent (pH >9 at 25°C) with 1.5 mM dNTP (PCR grade) and 3 mM MgCl2 (final concentrations) and a volume of 20 µl (mix 1). Reactions were carried out with a DNA Thermocycler System (MWG or MJ Research) with heated lid. To avoid sample evaporation, aqueous mixture in each tube was overlaid with 30–40 µl of mineral oil that was removed by chloroform extraction after the reaction.

After the first denaturation step at 98°C for 1 min, the thermal cycler was stopped at 85°C and 5 µl of diluted DNA polymerase (Takara LA Taq) was added. We diluted the polymerase 20-fold using 1× LA buffer. This step was followed by 30 cycles (98° for 10 s, 63°C for 1 min and 68°C for 6 to 12 min). After the PCR, DNA samples were precipitated with 2.5 vol of ethanol containing 2.5 M ammonium acetate at room temperature and no further purification was performed. Concentration of PCR product was measured using the NanoDrop spectrophotometer (NanoDrop, Wilmington, DE, USA) and verified by agarose gel electrophoresis.

The list of primers is available on Table S2. All the obtained Long-PCR amplicons were equimolarly mixed and subsequently split in two parts to use the same starting material for the different library preparation for Illumina GAII and Roche 454 GS FLX.

### Long-PCR Sequencing

GS FLX DNA Libraries were made following the manufacturer's protocol. Briefly, 4 µg of the 0.5 µM pooled amplicon solution was nebulized at 310 kPa for 1 min and purified using the MiniElute PCR purification kit (Qiagen). Small DNA fragments were removed using an AMPure PCR purification system (Agencourt Bioscience, Beverly, MA, USA) and analyzed on an Agilent 2100 Bioanalyzer. The nebulized DNA was subsequently end-repaired and phosphorylated using T4 DNA polymerase and T4 polynucleotide kinase, and oligonucleotide adapters were ligated to the DNA fragments with DNA ligase. Adapter-modified fragments were diluted, annealed to capture beads, and clonally amplified by emulsion PCR. After emulsion PCR, beads with clonal amplicons were enriched and deposited on a quarter of Picotiter plate flow cell and sequenced on a Roche 454 GS FLX platform.

Illumina's Paired-end libraries were made following the manufacturer's protocol with reagents supplied in the Illumina DNA sample kit. Briefly, 20 µl of the 0.5-µM pooled amplicon solution was nebulized at 220 kPa for 6 min. The nebulized DNA was end-repaired using Klenow and T4 DNA polymerases, phosphorylated with T4 polynucleotide kinase, and adenylated using Klenow exo-DNA polymerase, and oligonucleotide adapters were added using DNA ligase. Ligated products were visualized in a 2% agarose TBE gel, and a 200- to 250-bp size range was excised and purified using a Qiagen gel extraction kit. The size-selected, adapter-modified DNA fragments were amplified using adapter-specific primers 1.1 and 2.1 with Phusion DNA polymerase using the manufacturer's protocol. The library was purified prior to quantification using an Agilent 2100 Bioanalyzer (Agilent Technologies, Santa Clara, CA, USA). A single-flow cell lane was sequenced on a Illumina Genome Analyzer II. Confirmation of variants by Sanger sequencing was obtained using standard protocol. Sequences were run on ABI 3100 DNA analyzer, and assembled using ABI Prism Seqscape 2.1.

### Bioinformatic Sequence data analysis

All next-generation sequencing data were initially processed using the corresponding instrument software. Roche 454 data were initially processed using the GSMapper software package (Roche Inc.) supplied with the GS FLX instrument. High quality sequencing reads were aligned to the human genome reference sequence NCBI36/hg18. Variants with respect to NCBI36/hg18 reference sequence were identified with the Newbler software and the features obtained were remapped to GRCh37/hg19 using UCSC's liftOver tool (http://www.ncbi.nlm.nih.gov/genome/tools/remap) to be directly comparable with the other results. Illumina Genome Analyzer II data were initially processed using the Illumina Sequence Control Software (SCS) with Real Time Analysis (RTA) and Genome Analyzer Pipeline software supplied with the instrument. Sequence were aligned to the human genome reference sequence GRCh37 and variants were identified with using Illumina's Consensus Assessment of Sequence and Variation (CASAVA) software. SOLiD data were initially processed using the ICS software to obtain primary sequence analysis consisting of image analysis and basecalling colorspace fasta sequence. Color space reads were mapped to the GRCh37 reference genome with the SOLiD bioscope software v1.3 (reference) which utilizes an iterative mapping approach. Differences in the parameter used for the different chemistry used are available in Information S1. Single nucleotide variants were subsequently called by the DiBayes algorithm using the conservative default call stringency. Small insertions and deletions were detected using the SOLiD Small InDel Tool. Called SNP variants and indels were combined and annotated using a custom analysis pipeline. To spare software biases all the cross-platforms, comparison has been performed re-analyzing all the starting data using open-source software. Briefly the raw sequences obtained from each platform have been aligned to the human genome reference sequence GRCh37 using BWA [50] and quality filter before variant calling using Samtools software [51]. In the process of sequence variant calling, we focused only on variants included in coding exons and in corresponding canonical splicing sites of known Usher genes, considering as relevant for splicing sites investigations only the exons boundaries plus 2 base pairs. We utilized for further filtering our MYSQL in-house exome database, which includes sequencing data from 27 healthy individuals and 73 patients with non-ocular conditions. All the SNVs present in the database with an allele frequency >1% were not regarded as putative pathogenic mutations.

The Poliphen2 [52] software programs have been used to predict the influence of any amino acid substitution on the protein structure and function. PhastCons, and GERP have been used evaluate the conservation score of each variation. The pathogenic effect of missense variants was predicted as previously described [53] following a multi-step analysis [54] and is available in https://neuro-2.iurc.montp.inserm.fr/cgi-bin/USMA/USMA.fcgi. In addition, alignments of orthologs are accessible in USMA. Additional details are present as Information S1.

## Supporting Information

Figure S1 **Minimum coverage obtained with different enrichments methods.** Panel A shows the percentage of Usher exons sequenced based on the minimum coverage obtained with different enrichment methods. Solid colored lines represent mean values on three independent samples, dashed lines indicate the mean value+/−2 standard deviations from the mean. X axis indicates the minimum coverage increasing from left to right and is truncated at 50×. Y axis indicates the percentage of Usher exons basepair sequenced. Arbitrary threshold of 50% is represented using an horizontal dashed line. B–E) coverage in Usher related regions that fail in the following Exome enrichments: B, regions uncovered in SureSelect (50 MB); C, regions uncovered in Agilent SureSelectV2 (44 MB); D, regions uncovered in Agilent SureSelectV1 (38 MB); E, regions uncovered in TruSEq Exome. F) Boxplots of different Exome enrichments in regions that fail in Long-PCR enrichment, G) Boxplots of Long-PCR coverage for each Exome enrichments uncovered.
(TIF)

Figure S2 **Minimum coverage obtained for each Usher gene.** The figure shows base pair percentages of Usher exons sequenced based on the minimum coverage achieved on a gene-by-gene basis. Solid colored lines represent the mean values of three different samples processed using the same enrichment method. X axis indicates the minimum coverage increasing from left to right and is truncated at 50×. Y axis indicates the percentage of Usher exons basepair sequenced.
(TIF)

Figure S3 **Next Generation Sequencing whole exome and Long-PCR statistics.** A) Counts of sequence obtained from whole exome sequencing using Solid system and GAII or Roche GS FLX for Long-PCR. B) Counts of variations obtained in the Usher genomic regions before and after filter selection.
(TIF)

Figure S4 **Flowchart for whole exome process of screening and identifying variants.** All the data used in the Flowchart represent mean values of 9 independent samples.
(TIF)

Figure S5 **Next Generation Sequencing Long-PCR Sequencing statistics.** Statistic of on target base pairs obtained from Long-PCR Sequencing using GAII or Solid4 system.
(TIF)

Figure S6 **Long-PCR Coverage correlation.** The figure shows the nine Usher genes sequenced using Long-PCR in three independent samples and the coverage achieved with respect to the genomic position. X axis indicates the genomic position in base pairs and Y axis indicates the coverage. For each gene a legend table report the pair wise correlation value according Spearman's rank method confirming a strong position-dependent coverage correlation.
(TIF)

Figure S7 **Genomic regions of** ***MYO7A*** **variations.** A) The genomic position corresponding to variation *MYO7A* c.3827C>A shows a good score for primate, mammal and vertebrate conservation. Multi protein alignment shows the conservation of the corresponding S in 36 out of 46 vertebrate. B) The genomic position corresponding to variation *MYO7A* c.77C>A shows a good score for primate, mammal and vertebrate conservation. Multi protein alignment shows the conservation of the corresponding A in 41 out of 46 vertebrate.
(TIF)

Figure S8 **Reads coverage versus relative enrichment probe positions.** We considered only enrichment probes overlapping known Usher genes. All the data used for the graph represent mean values of three independent samples.. A) Agilent SureSelect Human all Exon v1(38 Mb) B) Agilent SureSelect Human all Exon v2(44 Mb) C) Agilent SureSelect Human all Exon (50 Mb) D) TrueSeq Exome (68 MB).
(TIF)

Figure S9 **Coverage distributions for different enrichment kits in the selected nine known Usher genes.** All the data used for the graph represent mean values of three independent samples. A) Agilent SureSelect Human all Exon v1(38 Mb) B) Agilent SureSelect Human all Exon v2(44 Mb) C) Agilent SureSelect Human all Exon (50 Mb) D) TrueSeq Exome (68 MB).
(TIF)

Information S1 **Supplementary** **Materials and Methods** **.**
(DOC)

Table S1 **List of sequence variants with a presumably pathogenic effect.**
(XLS)

Table S2 **Oligonucleotide primers used to generate long PCR products.**
(XLS)

Table S3 **Coverage and Strand bias test for USH patients Variants.**
(XLS)

## References

[1] Verpy E, Leibovici M, Zwaenepoel I, Liu XZ, Gal A, et al (2000) A defect in harmonin, a PDZ domain-containing protein expressed in the inner ear sensory hair cells, underlies Usher syndrome type 1C. Nat Genet 26: 51–55. 10973247 10.1038/79171

[2] Moller CG, Kimberling WJ, Davenport SL, Priluck I, White V, et al (1989) Usher syndrome: an otoneurologic study. Laryngoscope 99: 73–79. 2909824 10.1288/00005537-198901000-00014

[3] Hope CI, Bundey S, Proops D, Fielder AR (1997) Usher syndrome in the city of Birmingham–prevalence and clinical classification. Br J Ophthalmol 81: 46–53. 9135408 10.1136/bjo.81.1.46 PMC1721995

[4] Espinos C, Millan JM, Beneyto M, Najera C (1998) Epidemiology of Usher syndrome in Valencia and Spain. Community Genet 1: 223–228. 15178965 10.1159/000016167

[5] Spandau UH, Rohrschneider K (2002) Prevalence and geographical distribution of Usher syndrome in Germany. Graefes Arch Clin Exp Ophthalmol 240: 495–498. 12107518 10.1007/s00417-002-0485-8

[6] Millan JM, Aller E, Jaijo T, Blanco-Kelly F, Gimenez-Pardo A, et al (2011) An update on the genetics of usher syndrome. J Ophthalmol 2011: 417217. 21234346 10.1155/2011/417217 PMC3017948

[7] Yan D, Liu XZ (2010) Genetics and pathological mechanisms of Usher syndrome. J Hum Genet 55: 327–335. 20379205 10.1038/jhg.2010.29 PMC4511090

[8] Gibson F, Walsh J, Mburu P, Varela A, Brown KA, et al (1995) A type VII myosin encoded by the mouse deafness gene shaker-1. Nature 374: 62–64. 7870172 10.1038/374062a0

[9] Weil D, Blanchard S, Kaplan J, Guilford P, Gibson F, et al (1995) Defective myosin VIIA gene responsible for Usher syndrome type 1B. Nature 374: 60–61. 7870171 10.1038/374060a0

[10] Bonnet C, Grati M, Marlin S, Levilliers J, Hardelin JP, et al (2011) Complete exon sequencing of all known Usher syndrome genes greatly improves molecular diagnosis. Orphanet J Rare Dis 6: 21. 21569298 10.1186/1750-1172-6-21 PMC3125325

[11] Weston MD, Kelley PM, Overbeck LD, Wagenaar M, Orten DJ, et al (1996) Myosin VIIA mutation screening in 189 Usher syndrome type 1 patients. Am J Hum Genet 59: 1074–1083. 8900236 PMC1914835

[12] Astuto LM, Weston MD, Carney CA, Hoover DM, Cremers CW, et al (2000) Genetic heterogeneity of Usher syndrome: analysis of 151 families with Usher type I. Am J Hum Genet 67: 1569–1574. 11060213 10.1086/316889 PMC1287932

[13] Le Quesne Stabej P, Saihan Z, Rangesh N, Steele-Stallard HB, Ambrose J, et al (2011) Comprehensive sequence analysis of nine Usher syndrome genes in the UK National Collaborative Usher Study. J Med Genet 49: 27–36. 22135276 10.1136/jmedgenet-2011-100468 PMC3678402

[14] Bolz H, von Brederlow B, Ramirez A, Bryda EC, Kutsche K, et al (2001) Mutation of CDH23, encoding a new member of the cadherin gene family, causes Usher syndrome type 1D. Nat Genet 27: 108–112. 11138009 10.1038/83667

[15] Bork JM, Peters LM, Riazuddin S, Bernstein SL, Ahmed ZM, et al (2001) Usher syndrome 1D and nonsyndromic autosomal recessive deafness DFNB12 are caused by allelic mutations of the novel cadherin-like gene CDH23. Am J Hum Genet 68: 26–37. 11090341 10.1086/316954 PMC1234923

[16] Alagramam KN, Yuan H, Kuehn MH, Murcia CL, Wayne S, et al (2001) Mutations in the novel protocadherin PCDH15 cause Usher syndrome type 1F. Hum Mol Genet 10: 1709–1718. 11487575 10.1093/hmg/10.16.1709

[17] Ahmed ZM, Riazuddin S, Bernstein SL, Ahmed Z, Khan S, et al (2001) Mutations of the protocadherin gene PCDH15 cause Usher syndrome type 1F. Am J Hum Genet 69: 25–34. 11398101 10.1086/321277 PMC1226045

[18] Ouyang XM, Yan D, Du LL, Hejtmancik JF, Jacobson SG, et al (2005) Characterization of Usher syndrome type I gene mutations in an Usher syndrome patient population. Hum Genet 116: 292–299. 15660226 10.1007/s00439-004-1227-2

[19] Bitner-Glindzicz M, Lindley KJ, Rutland P, Blaydon D, Smith VV, et al (2000) A recessive contiguous gene deletion causing infantile hyperinsulinism, enteropathy and deafness identifies the Usher type 1C gene. Nat Genet 26: 56–60. 10973248 10.1038/79178

[20] Kikkawa Y, Shitara H, Wakana S, Kohara Y, Takada T, et al (2003) Mutations in a new scaffold protein Sans cause deafness in Jackson shaker mice. Hum Mol Genet 12: 453–461. 12588793 10.1093/hmg/ddg042

[21] Iannaccone A, Kritchevsky SB, Ciccarelli ML, Tedesco SA, Macaluso C, et al (2004) Kinetics of visual field loss in Usher syndrome Type II. Invest Ophthalmol Vis Sci 45: 784–792. 14985291 10.1167/iovs.03-0906

[22] Pieke-Dahl S, van Aarem A, Dobin A, Cremers CW, Kimberling WJ (1996) Genetic heterogeneity of Usher syndrome type II in a Dutch population. J Med Genet 33: 753–757. 8880575 10.1136/jmg.33.9.753 PMC1050729

[23] Weston MD, Eudy JD, Fujita S, Yao S, Usami S, et al (2000) Genomic structure and identification of novel mutations in usherin, the gene responsible for Usher syndrome type IIa. Am J Hum Genet 66: 1199–1210. 10729113 10.1086/302855 PMC1288187

[24] Weston MD, Luijendijk MW, Humphrey KD, Moller C, Kimberling WJ (2004) Mutations in the VLGR1 gene implicate G-protein signaling in the pathogenesis of Usher syndrome type II. Am J Hum Genet 74: 357–366. 14740321 10.1086/381685 PMC1181933

[25] Ebermann I, Scholl HP, Charbel Issa P, Becirovic E, Lamprecht J, et al (2007) A novel gene for Usher syndrome type 2: mutations in the long isoform of whirlin are associated with retinitis pigmentosa and sensorineural hearing loss. Hum Genet 121: 203–211. 17171570 10.1007/s00439-006-0304-0

[26] Mburu P, Mustapha M, Varela A, Weil D, El-Amraoui A, et al (2003) Defects in whirlin, a PDZ domain molecule involved in stereocilia elongation, cause deafness in the whirler mouse and families with DFNB31. Nat Genet 34: 421–428. 12833159 10.1038/ng1208

[27] Adato A, Vreugde S, Joensuu T, Avidan N, Hamalainen R, et al (2002) USH3A transcripts encode clarin-1, a four-transmembrane-domain protein with a possible role in sensory synapses. Eur J Hum Genet 10: 339–350. 12080385 10.1038/sj.ejhg.5200831

[28] Joensuu T, Hamalainen R, Yuan B, Johnson C, Tegelberg S, et al (2001) Mutations in a novel gene with transmembrane domains underlie Usher syndrome type 3. Am J Hum Genet 69: 673–684. 11524702 10.1086/323610 PMC1226054

[29] Cremers FP, Kimberling WJ, Kulm M, de Brouwer AP, van Wijk E, et al (2007) Development of a genotyping microarray for Usher syndrome. J Med Genet 44: 153–160. 16963483 10.1136/jmg.2006.044784 PMC2598068

[30] Jaijo T, Aller E, Garcia-Garcia G, Aparisi MJ, Bernal S, et al (2009) Microarray-based mutation analysis of 183 Spanish families with Usher syndrome. Invest Ophthalmol Vis Sci 51: 1311–1317. 19683999 10.1167/iovs.09-4085

[31] Vozzi D, Aaspollu A, Athanasakis E, Berto A, Fabretto A, et al (2011) Molecular epidemiology of Usher syndrome in Italy. Mol Vis 17: 1662–1668. 21738395 PMC3130723

[32] Chepelev I, Wei G, Tang Q, Zhao K (2009) Detection of single nucleotide variations in expressed exons of the human genome using RNA-Seq. Nucleic Acids Res 37: e106. 19528076 10.1093/nar/gkp507 PMC2760790

[33] Schuster SC (2008) Next-generation sequencing transforms today's biology. Nat Methods 5: 16–18. 18165802 10.1038/nmeth1156

[34] Margulies M, Egholm M, Altman WE, Attiya S, Bader JS, et al (2005) Genome sequencing in microfabricated high-density picolitre reactors. Nature 437: 376–380. 16056220 10.1038/nature03959 PMC1464427

[35] Ng SB, Buckingham KJ, Lee C, Bigham AW, Tabor HK, et al (2009) Exome sequencing identifies the cause of a mendelian disorder. Nat Genet 42: 30–35. 19915526 10.1038/ng.499 PMC2847889

[36] Roach JC, Glusman G, Smit AF, Huff CD, Hubley R, et al (2010) Analysis of genetic inheritance in a family quartet by whole-genome sequencing. Science 328: 636–639. 20220176 10.1126/science.1186802 PMC3037280

[37] Hoischen A, van Bon BW, Gilissen C, Arts P, van Lier B, et al (2010) De novo mutations of SETBP1 cause Schinzel-Giedion syndrome. Nat Genet 42: 483–485. 20436468 10.1038/ng.581

[38] Lupski JR, Reid JG, Gonzaga-Jauregui C, Rio Deiros D, Chen DC, et al (2010) Whole-genome sequencing in a patient with Charcot-Marie-Tooth neuropathy. N Engl J Med 362: 1181–1191. 20220177 10.1056/NEJMoa0908094 PMC4036802

[39] Ng SB, Turner EH, Robertson PD, Flygare SD, Bigham AW, et al (2009) Targeted capture and massively parallel sequencing of 12 human exomes. Nature 461: 272–276. 19684571 10.1038/nature08250 PMC2844771

[40] Voelkerding KV, Dames S, Durtschi JD (2010) Next generation sequencing for clinical diagnostics-principles and application to targeted resequencing for hypertrophic cardiomyopathy: a paper from the 2009 William Beaumont Hospital Symposium on Molecular Pathology. J Mol Diagn 12: 539–551. 20805560 10.2353/jmoldx.2010.100043 PMC2928417

[41] Bonnal RJ, Severgnini M, Castaldi A, Bordoni R, Iacono M, et al (2010) Reliable resequencing of the human dystrophin locus by universal long polymerase chain reaction and massive pyrosequencing. Anal Biochem 406: 176–184. 20670611 10.1016/j.ab.2010.07.022

[42] Chou LS, Liu CS, Boese B, Zhang X, Mao R (2009) DNA sequence capture and enrichment by microarray followed by next-generation sequencing for targeted resequencing: neurofibromatosis type 1 gene as a model. Clin Chem 56: 62–72. 19910506 10.1373/clinchem.2009.132639

[43] Neveling K, Collin RW, Gilissen C, van Huet RA, Visser L, et al (2012) Next-generation genetic testing for retinitis pigmentosa. Hum Mutat 33: 963–972. 22334370 10.1002/humu.22045 PMC3490376

[44] Yeager M, Xiao N, Hayes RB, Bouffard P, Desany B, et al (2008) Comprehensive resequence analysis of a 136 kb region of human chromosome 8q24 associated with prostate and colon cancers. Hum Genet 124: 161–170. 18704501 10.1007/s00439-008-0535-3 PMC2525844

[45] Harismendy O, Ng PC, Strausberg RL, Wang X, Stockwell TB, et al (2009) Evaluation of next generation sequencing platforms for population targeted sequencing studies. Genome Biol 10: R32. 19327155 10.1186/gb-2009-10-3-r32 PMC2691003

[46] Morozova O, Marra MA (2008) Applications of next-generation sequencing technologies in functional genomics. Genomics 92: 255–264. 18703132 10.1016/j.ygeno.2008.07.001

[47] Summerer D (2009) Enabling technologies of genomic-scale sequence enrichment for targeted high-throughput sequencing. Genomics 94: 363–368. 19720138 10.1016/j.ygeno.2009.08.012

[48] Wei X, Ju X, Yi X, Zhu Q, Qu N, et al (2011) Identification of sequence variants in genetic disease-causing genes using targeted next-generation sequencing. PLoS One 6: e29500. 22216297 10.1371/journal.pone.0029500 PMC3244462

[49] Astuto LM, Bork JM, Weston MD, Askew JW, Fields RR, et al (2002) CDH23 mutation and phenotype heterogeneity: a profile of 107 diverse families with Usher syndrome and nonsyndromic deafness. Am J Hum Genet 71: 262–275. 12075507 10.1086/341558 PMC379159

[50] Li H, Durbin R (2009) Fast and accurate short read alignment with Burrows-Wheeler transform. Bioinformatics 25: 1754–1760. 19451168 10.1093/bioinformatics/btp324 PMC2705234

[51] Li H, Handsaker B, Wysoker A, Fennell T, Ruan J, et al (2009) The Sequence Alignment/Map format and SAMtools. Bioinformatics 25: 2078–2079. 19505943 10.1093/bioinformatics/btp352 PMC2723002

[52] Adzhubei IA, Schmidt S, Peshkin L, Ramensky VE, Gerasimova A, et al (2010) A method and server for predicting damaging missense mutations. Nat Methods 7: 248–249. 20354512 10.1038/nmeth0410-248 PMC2855889

[53] Baux D, Larrieu L, Blanchet C, Hamel C, Ben Salah S, et al (2007) Molecular and in silico analyses of the full-length isoform of usherin identify new pathogenic alleles in Usher type II patients. Hum Mutat 28: 781–789. 17405132 10.1002/humu.20513

[54] Le Guedard-Mereuze S, Vache C, Baux D, Faugere V, Larrieu L, et al (2010) Ex vivo splicing assays of mutations at noncanonical positions of splice sites in USHER genes. Hum Mutat 31: 347–355. 20052763 10.1002/humu.21193

