# Supplementary figures and images for: Molecular Diagnosis of Usher Syndrome: Application of Two Different Next Generation Sequencing-Based Procedures
Source: PLoS One. 2012 Aug 29;7(8):e43799. doi: 10.1371/journal.pone.0043799 (PMC3430670; doi:10.1371/journal.pone.0043799)

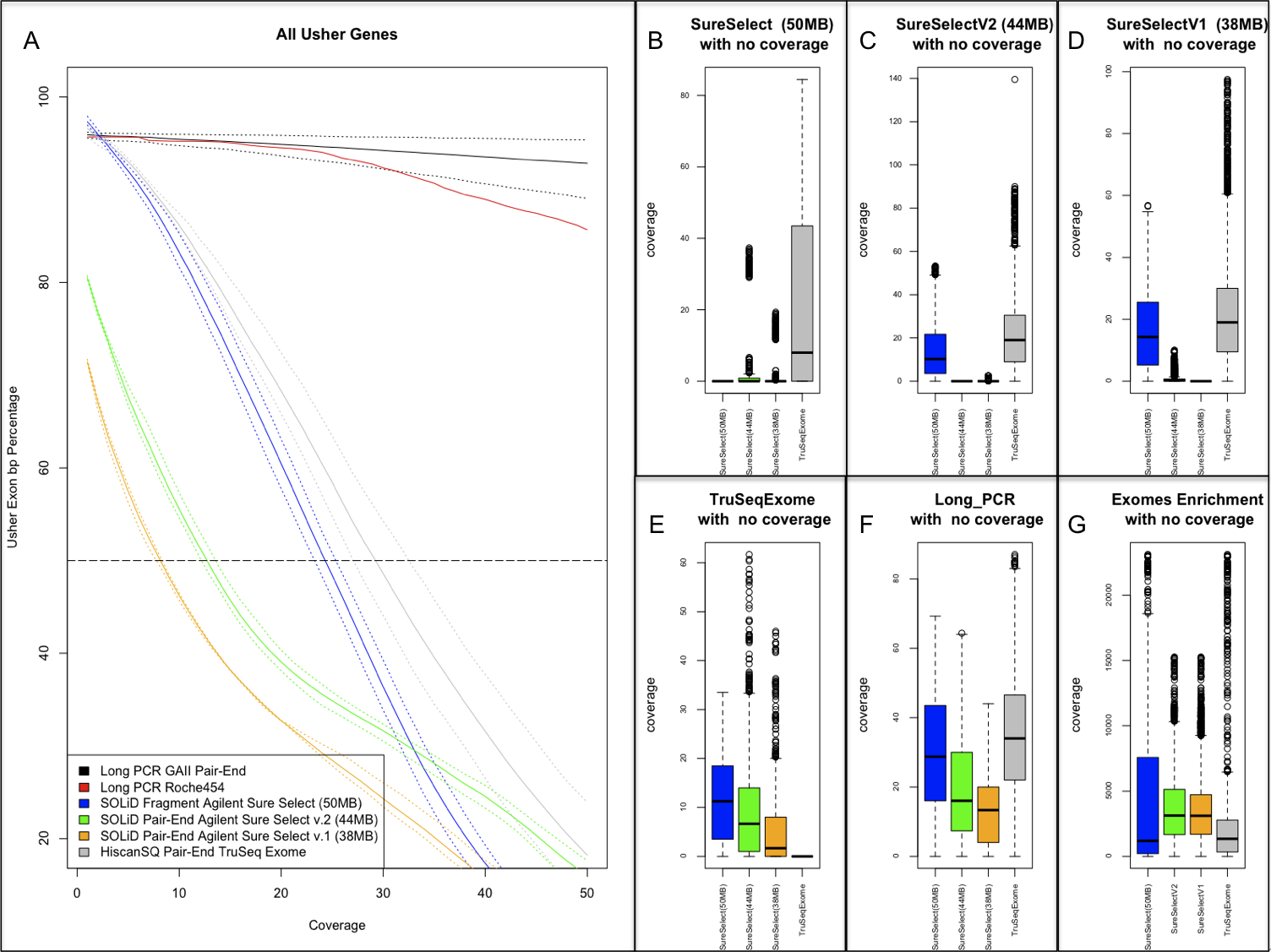

Supplement: Figure S1 — Minimum coverage obtained with different enrichments methods. Panel A shows the percentage of Usher exons sequenced based on the minimum coverage obtained with different enrichment methods. Solid colored lines represent mean values on three independent samples, dashed lines indicate the mean value+/−2 standard deviations from the mean. X axis indicates the minimum coverage increasing from left to right and is truncated at 50×. Y axis indicates the percentage of Usher exons basepair sequenced. Arbitrary threshold of 50% is represented using an horizontal dashed line. B–E) coverage in Usher related regions that fail in the following Exome enrichments: B, regions uncovered in SureSelect (50 MB); C, regions uncovered in Agilent SureSelectV2 (44 MB); D, regions uncovered in Agilent SureSelectV1 (38 MB); E, regions uncovered in TruSEq Exome. F) Boxplots of different Exome enrichments in regions that fail in Long-PCR enrichment, G) Boxplots of Long-PCR coverage for each Exome enrichments uncovered. (TIF) [file pone.0043799.s001.tif]

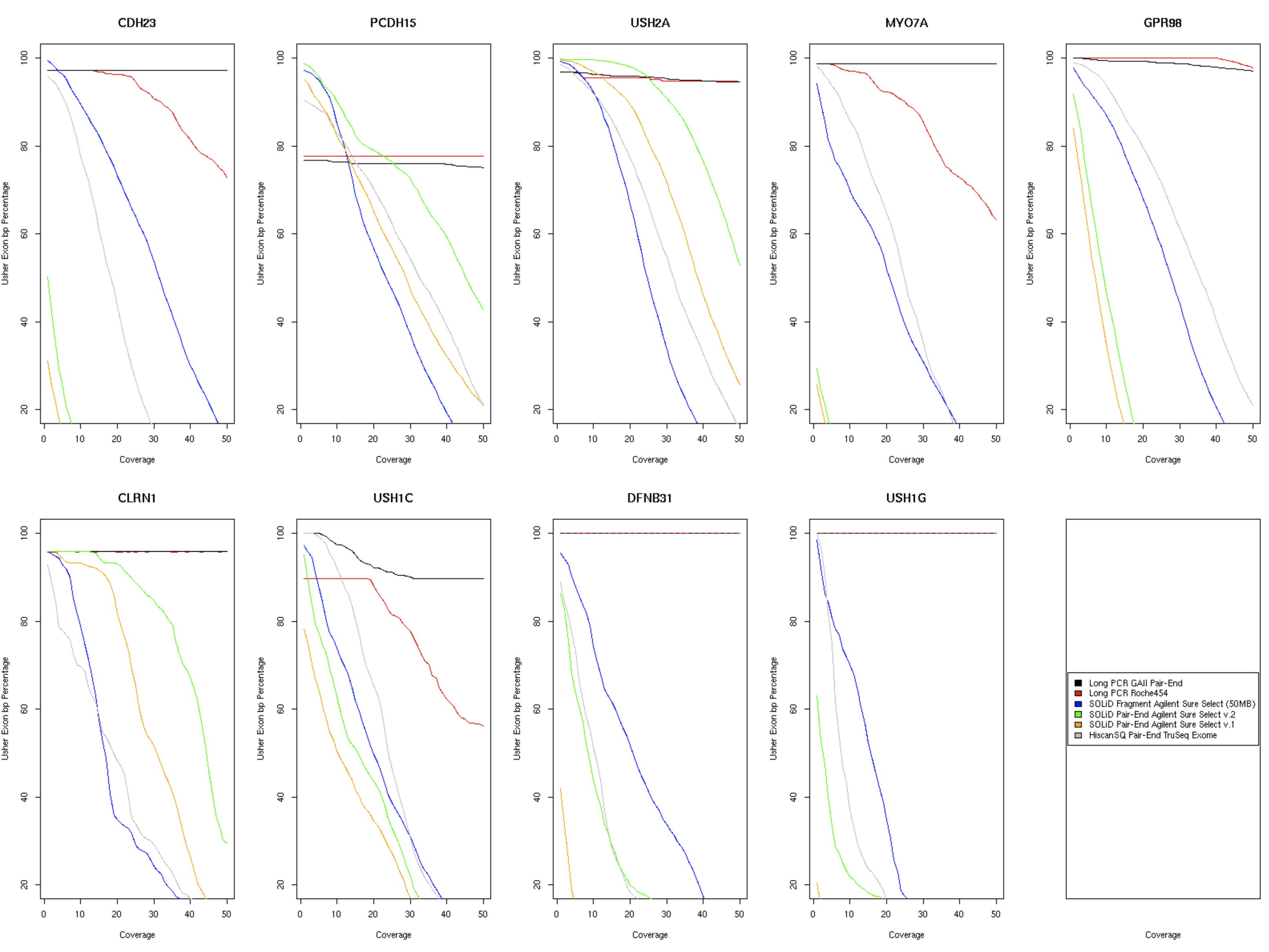

Supplement: Figure S2 — Minimum coverage obtained for each Usher gene. The figure shows base pair percentages of Usher exons sequenced based on the minimum coverage achieved on a gene-by-gene basis. Solid colored lines represent the mean values of three different samples processed using the same enrichment method. X axis indicates the minimum coverage increasing from left to right and is truncated at 50×. Y axis indicates the percentage of Usher exons basepair sequenced. (TIF) [file pone.0043799.s002.tif]

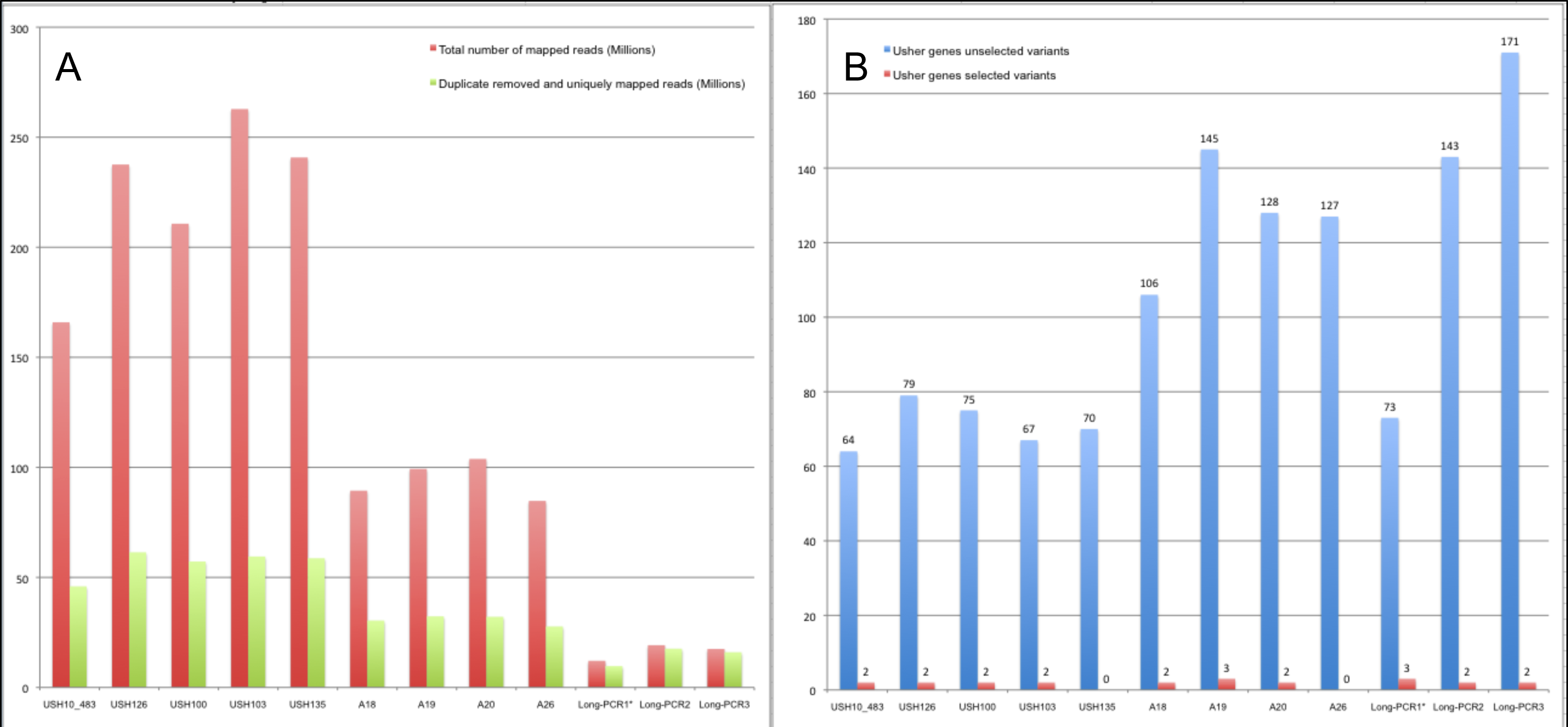

Supplement: Figure S3 — Next Generation Sequencing whole exome and Long-PCR statistics. A) Counts of sequence obtained from whole exome sequencing using Solid system and GAII or Roche GS FLX for Long-PCR. B) Counts of variations obtained in the Usher genomic regions before and after filter selection. (TIF) [file pone.0043799.s003.tif]

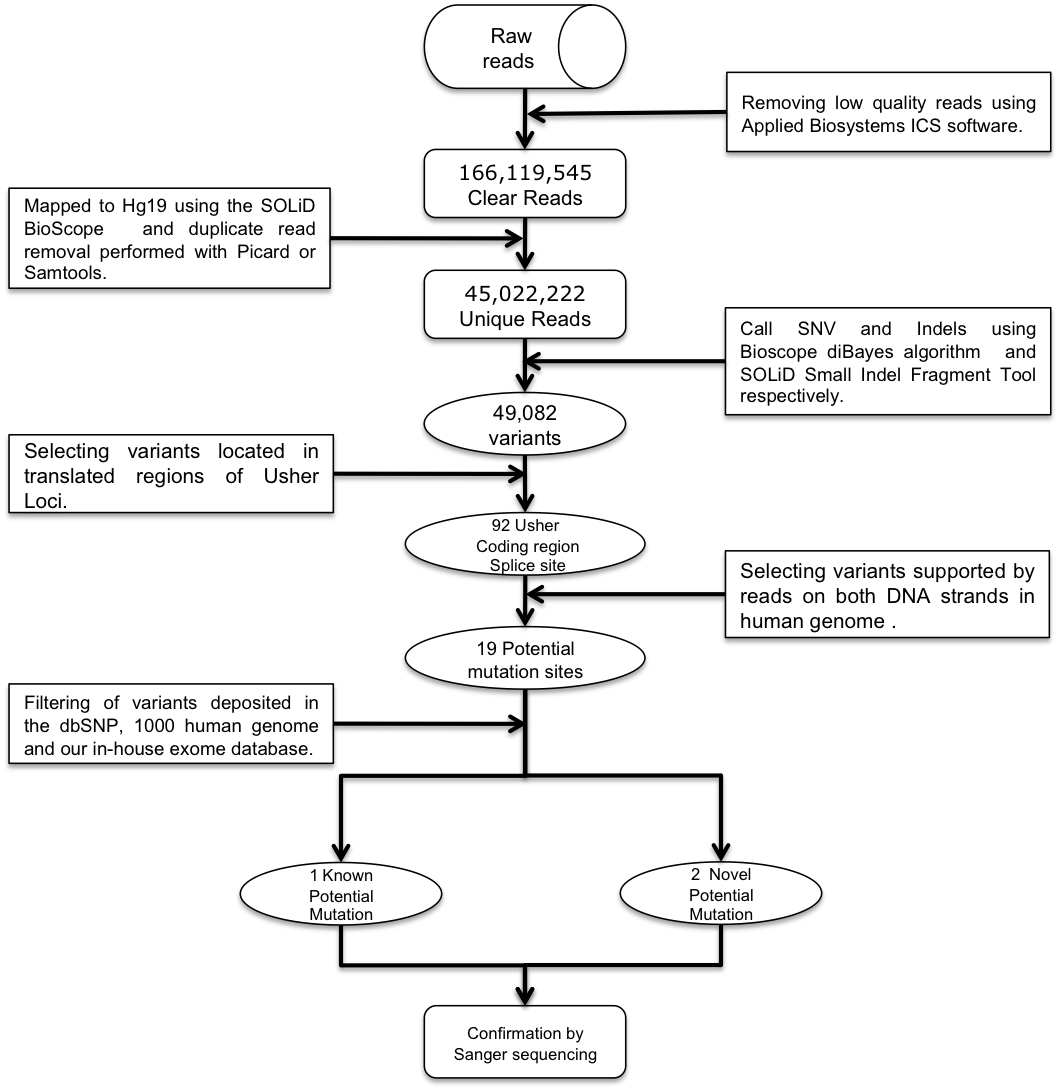

Supplement: Figure S4 — Flowchart for whole exome process of screening and identifying variants. All the data used in the Flowchart represent mean values of 9 independent samples. (TIF) [file pone.0043799.s004.tif]

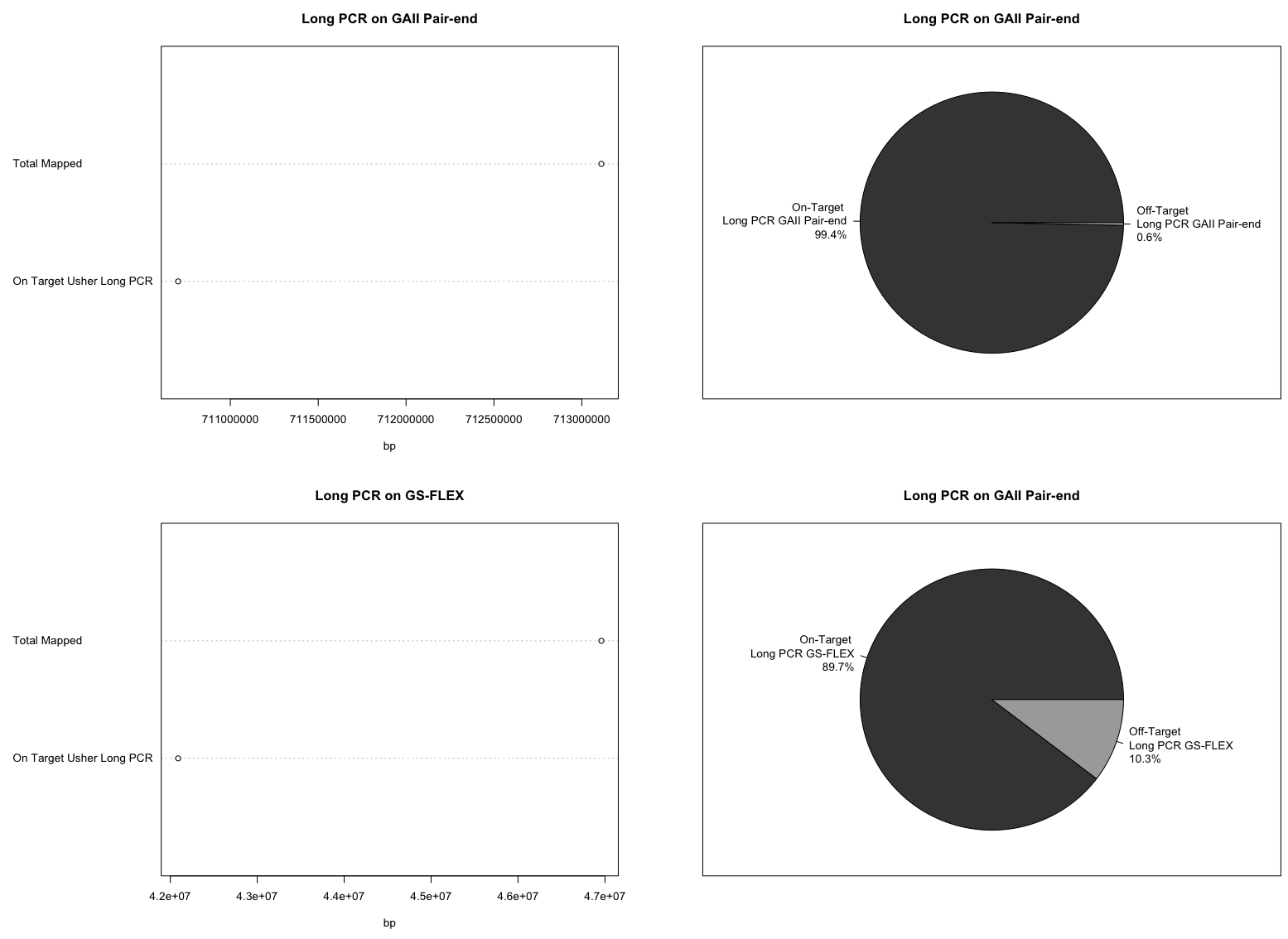

Supplement: Figure S5 — Next Generation Sequencing Long-PCR Sequencing statistics. Statistic of on target base pairs obtained from Long-PCR Sequencing using GAII or Solid4 system. (TIF) [file pone.0043799.s005.tif]

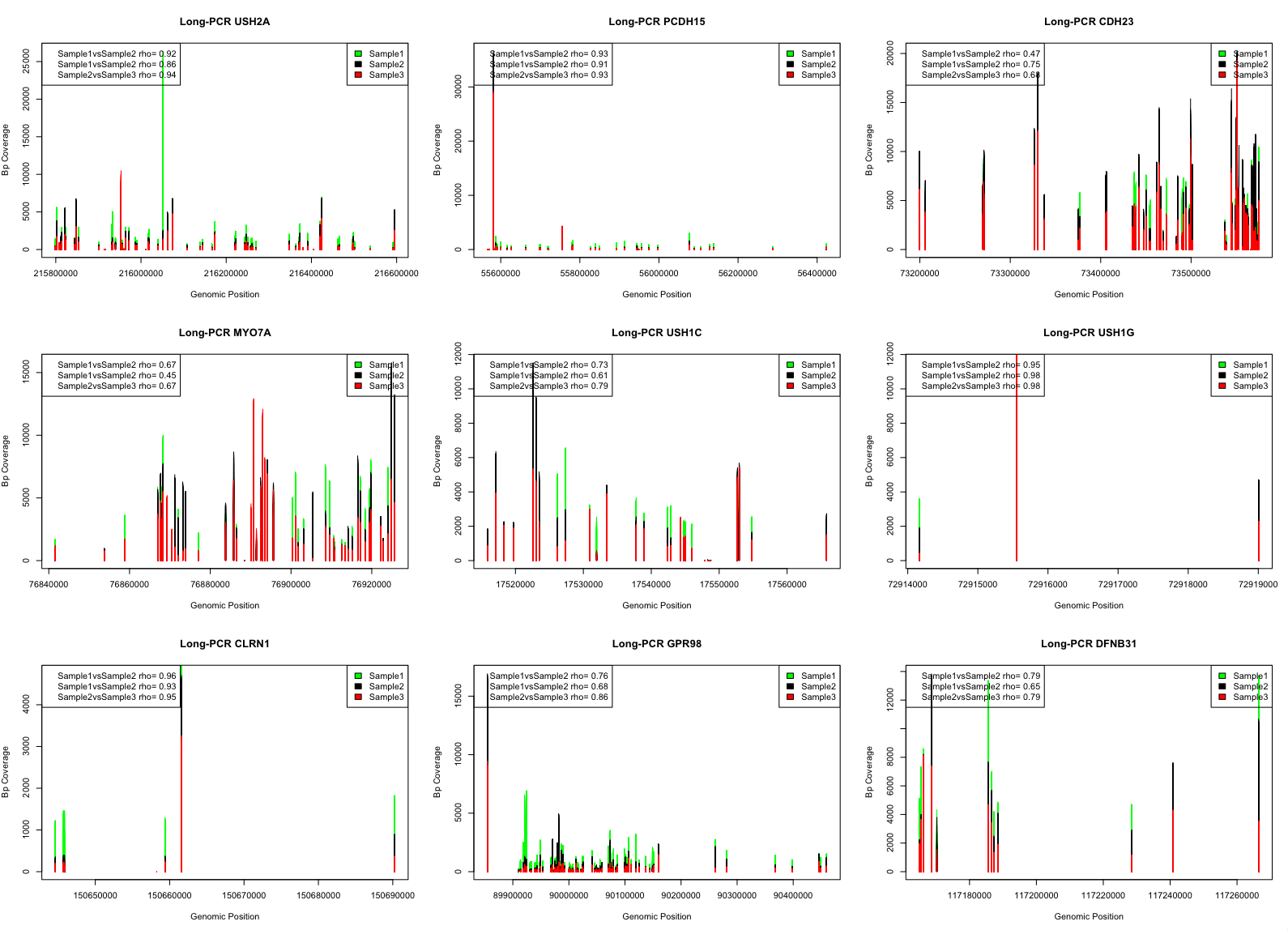

Supplement: Figure S6 — Long-PCR Coverage correlation. The figure shows the nine Usher genes sequenced using Long-PCR in three independent samples and the coverage achieved with respect to the genomic position. X axis indicates the genomic position in base pairs and Y axis indicates the coverage. For each gene a legend table report the pair wise correlation value according Spearman's rank method confirming a strong position-dependent coverage correlation. (TIF) [file pone.0043799.s006.tif]

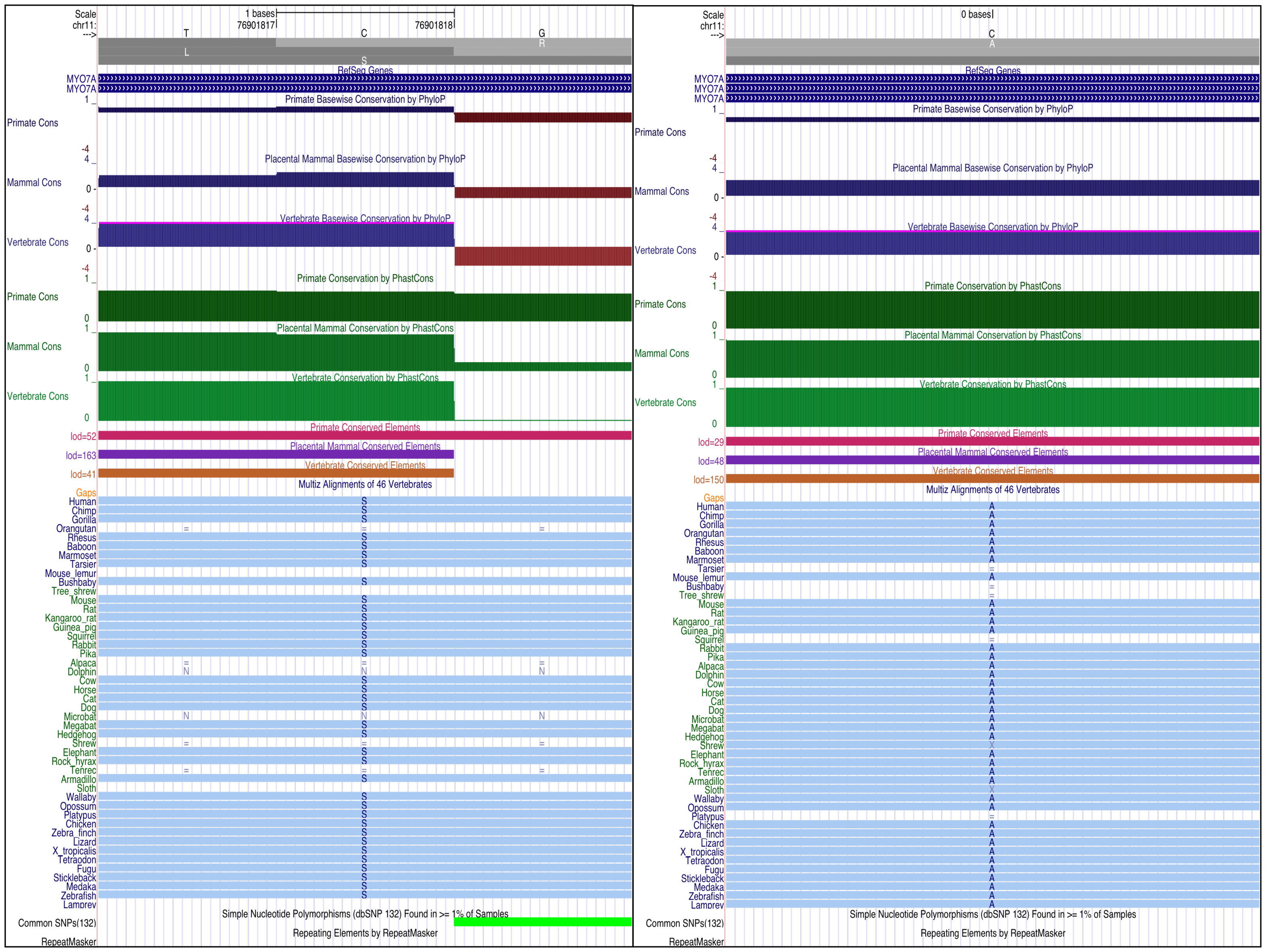

Supplement: Figure S7 — Genomic regions of MYO7A variations. A) The genomic position corresponding to variation MYO7A c.3827C>A shows a good score for primate, mammal and vertebrate conservation. Multi protein alignment shows the conservation of the corresponding S in 36 out of 46 vertebrate. B) The genomic position corresponding to variation MYO7A c.77C>A shows a good score for primate, mammal and vertebrate conservation. Multi protein alignment shows the conservation of the corresponding A in 41 out of 46 vertebrate. (TIF) [file pone.0043799.s007.tif]

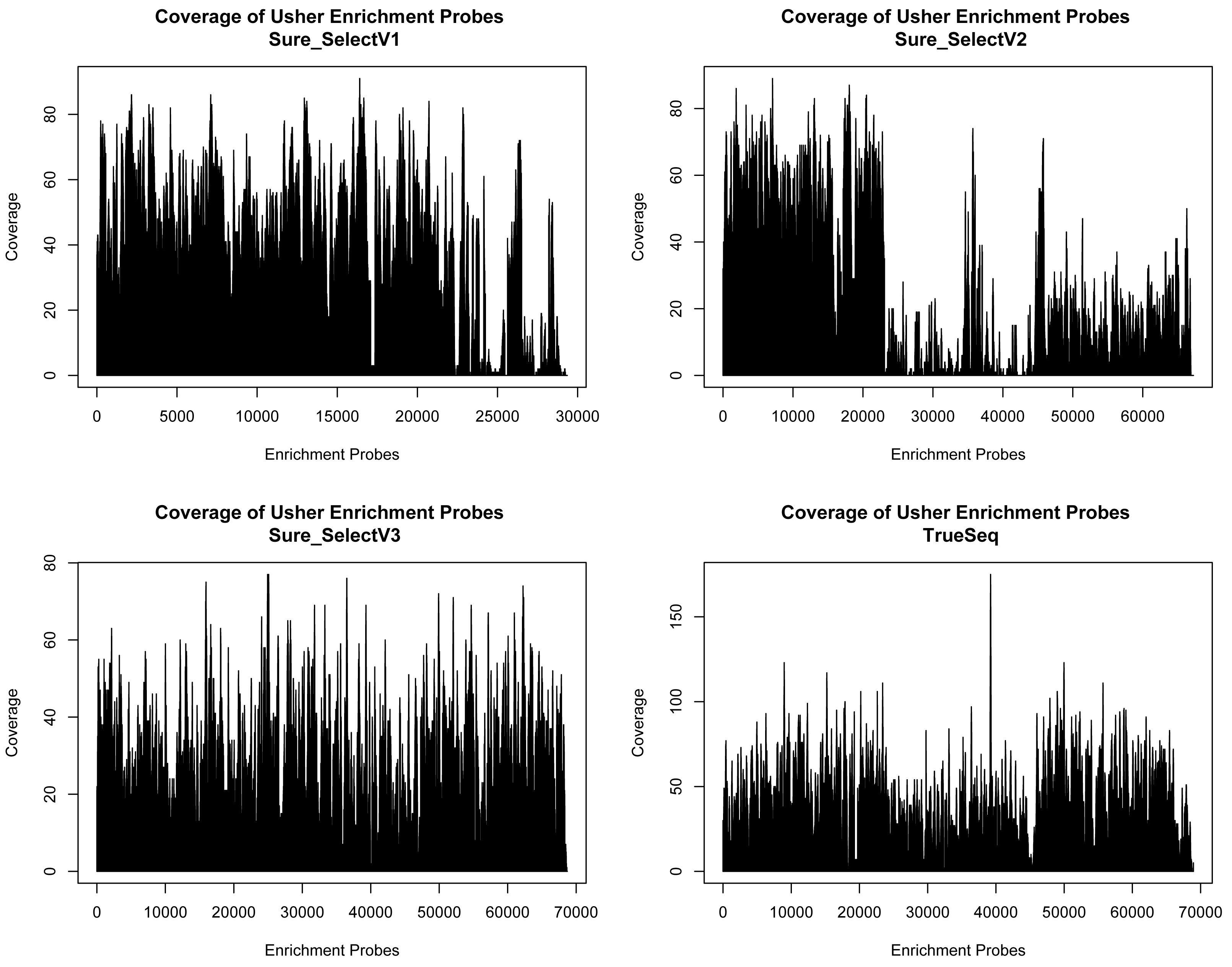

Supplement: Figure S8 — Reads coverage versus relative enrichment probe positions. We considered only enrichment probes overlapping known Usher genes. All the data used for the graph represent mean values of three independent samples.. A) Agilent SureSelect Human all Exon v1(38 Mb) B) Agilent SureSelect Human all Exon v2(44 Mb) C) Agilent SureSelect Human all Exon (50 Mb) D) TrueSeq Exome (68 MB). (TIF) [file pone.0043799.s008.tif]

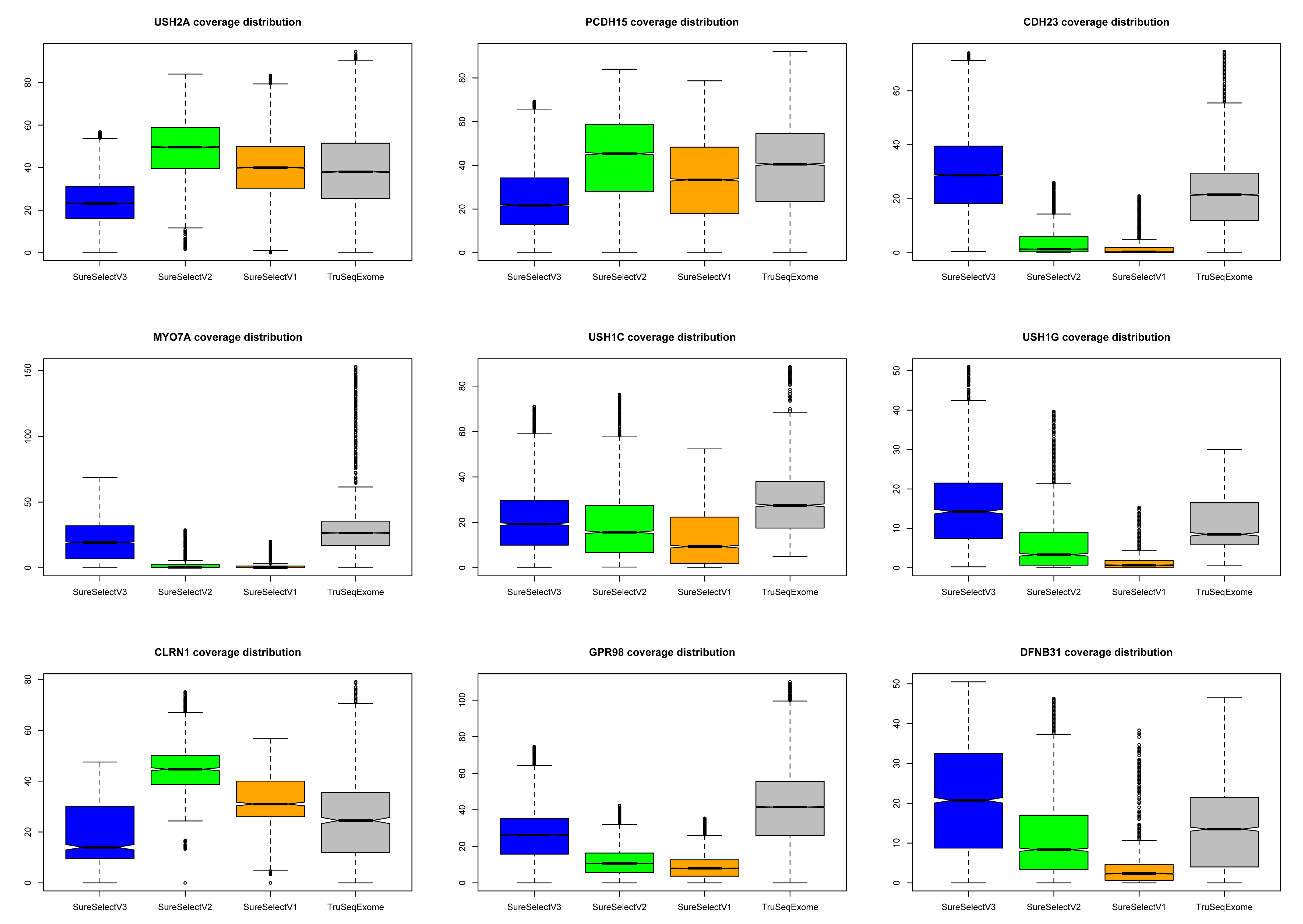

Supplement: Figure S9 — Coverage distributions for different enrichment kits in the selected nine known Usher genes. All the data used for the graph represent mean values of three independent samples. A) Agilent SureSelect Human all Exon v1(38 Mb) B) Agilent SureSelect Human all Exon v2(44 Mb) C) Agilent SureSelect Human all Exon (50 Mb) D) TrueSeq Exome (68 MB). (TIF) [file pone.0043799.s009.tif]
