# Supplementary material for: Molecular Diagnosis of Usher Syndrome: Application of Two Different Next Generation Sequencing-Based Procedures
Source: PLoS One. 2012 Aug 29;7(8):e43799. doi: 10.1371/journal.pone.0043799 (PMC3430670; doi:10.1371/journal.pone.0043799)
Supplement: Information S1 — Supplementary Materials and Methods . (DOC) [file pone.0043799.s010.doc]

**Supporting Information.**

Molecular diagnosis of Usher syndrome: evaluation of two different Next Generation Sequencing-based procedures

**D. Licastro^1^, M. Mutarelli^2^, I. Peluso^2^, K. Neveling^3^, N. Wieskamp^3^,R. Rispoli^2^, D. Vozzi^4^, E. Athanasakis^4^, A. D’Eustacchio^4^, M. Pizzo^2^, F. D’Amico^5^, C. Ziviello^2^, F. Simonelli^6^, A. Fabretto^4^, H. Scheffer^3^, P. Gasparini^4^***^§^,* **S. Banfi^2,5^***^§^,* **V. Nigro^5^***^§^*

*^1^Cluster in Biomedicine (CBM) scrl - Genomics, Area Science Park, Basovizza, Trieste, Italy.*

*^2^Telethon Institute of Genetics and Medicine (TIGEM), via Pietro Castellino 111, 80131, Napoli, Italy*

*^3^ Radboud University Nijmegen Medical Center, Geert Grooteplein 10, 6525 GA Nijmegen, Netherlands.*

*^4^ Institute for Maternal and Child Health - IRCCS “Burlo Garofolo”.*

*^5^ Seconda Università degli Studi di Napoli-Dip. Patologia Generale,Via Luigi De Crecchio 7, 80138 Napoli*

*^6^Department of Ophthalmology, Seconda Università degli Studi di Napoli, Naples, Italy*

*^§^ Correspondence should be addressed to SB, VN (email:* *banfi@tigem.it,* *gasparini@burlo.trieste.it,vincenzo.nigro@unina2.it )*

### SOLiD Exome sequencing.

For Agilent exome enrichment 3µg genomic DNA was required. We used AB SOLiD optimized kits (Agilent, Santa Clara, CA, USA), following the manufacturer's instructions. Briefly for every 3 µg DNA, we diluted the genomic DNA and, using a Covaris station, sheered the genomic DNA to 150 base pair. The purified obtained sample were ends repaired, adaptor legated and the obtained library amplified according to SureSelect Target Enrichment protocol. The final step of Hybrid Capture Selection provided an Enriched library that has been quality assessed with Agilent 2100 Bioanalyzer. The enriched exome libraries were subsequently used for e-PCRs following manufacturer's instructions (Life Technologies, Carlsbad, CA, USA), based on a library concentration of 0.5pM. Sequencing was performed using SOLiD3 or SOLiD4 system that involves ligation-based sequencing and a two-base encoding method in which four fluorescent dyes are used to tag various combinations of dinucle tides. Its accuracy in sequencing is estimated at approximately 99.94%. SOLiD data were initially processed using the Applied Biosystems ICS software to obtain primary sequence analysis consisting of image analysis and base‐calling colorspace fasta sequences with quality scores. Color space reads were first color-corrected using SOLiD Accuracy Enhancer Tool (SAET). The corrected reads were mapped to the GRCh37 reference genome with the SOLiD BioScope software v1.3 [1] with the local mapping mode and utilizing and iterative mapping approach. After duplicate read removal performed with Picard [2] or Samtools, single nucleotide variants were called with the diBayes algorithm [3] using medium stringency settings. Small insertions and deletions were detected using the SOLiD Small Indel Fragment Tool [1]. Called single nucleotide variants and in-dels were combined, annotated and inserted in a local variation database using a custom analysis pipeline. Annotation was performed with ANNOVAR [4], including the relative position in genes using RefSeq [5] gene model, amminoacid change, presence in dbSNP[6], frequency in 1000 genomes samples [7], conservation and different prediction scores of protein damage [8, 9, 10,11].Specific changes for 50bp fragment chemistry and 50+35bp pair-end data are reported in the following table.


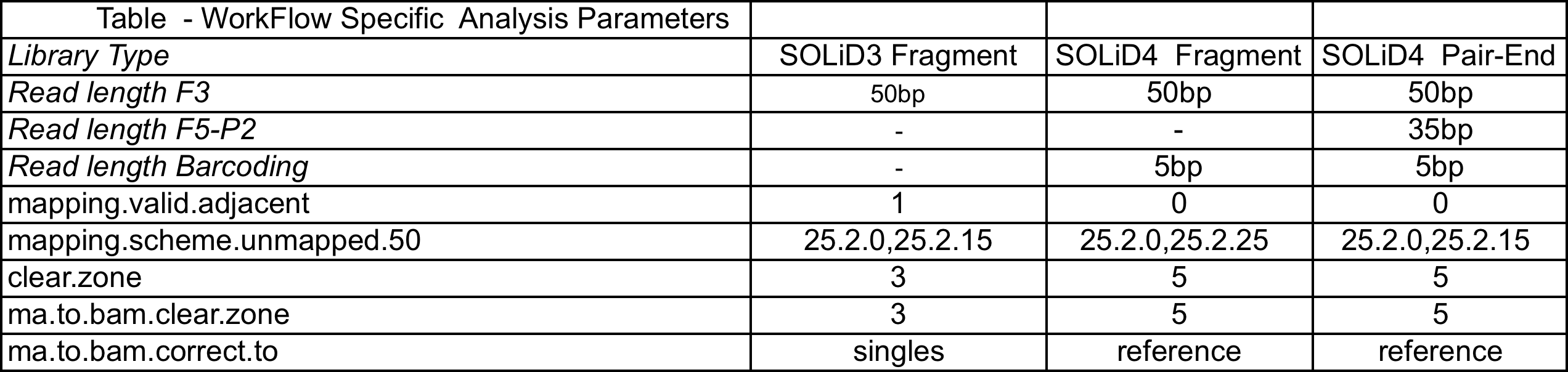


### TruSeq Exome Enrichment on HiseqSQ.

For exome enrichment 1µg of genomic DNA was required as starting matherial an enriched with Illumina TruSeq Exome Enrichment kit, which inludes more than 340.000 95mer probes targeting exonic sequences of ~20.794 genes.While the sum length of these probes is 32M, the kit actually targets 62Mb of the human genome. Briefly for every 1µg of genomic DNA a human DNA library was prepared using the Illumina® TruSeqTM DNA Sample Prep Kit , following the manufacturer's instructions. For every 1 µg DNA, we dilute the genomic DNA and using a Covaris station we sheered the genomic DNA to 300 base pair. The purified obtained sample has been ends repaired, 3’ adenilated nd adaptor ligated using the the appropriate DNA Adapter Index tubes.The obtained library has been PCR amplified to selectively enrich those DNA fragments that have adapter molecole on both ends.The final purified product has been quantitated using both qPCR and Agilent 2100 Bioanalyzer (Agilent), 500 ng of each DNA library has been used to exome capture after pooling of 4 librares. Data from TruSeq Exome Enrichment has been used only for the Different Enrichment approaches comparison.

***Different Enrichment approaches show different coverage for the 9 Usher genes.***

For this analysis, we used four different commercial in solution Exome Enrichment Kits prior to high throughput sequencing. In the analysis we focused only on the coding region of the nine genes known to be involved in Usher Syndrome. Our comparison (Figure S1) clearly shows that even considering a minimum coverage of 1 the percentage of coding base pair sequenced range from a maximum of 97% obtained with the Agilent SureSelect 50MB and Illumina TrueSeq Exome enrichment, to a minimum of 71% obtained with the Agilent SureSelect V1.0 showing that variability of these method reflect exactly the extent of overlap between enrichment probes and exons. In a whole exome enrichment the exons of Usher genes represent only a small portion of the enrichment (Figure S4) and, as expected, the percentage of sequenced regions drops upon increasing the depth demand on all the Enrichment methods but the slope is less severe in the case of a Long-PCR approach compared with any Whole Exome Sequencing (Figure S1, left panel). Interestingly considering a threshold of 50% Usher Exons bp sequenced, we can push the system to a maximun coverage of 8x for Agilent SureSelect 38Mb, 13x for Agilent SureSelect 48Mb and a 24x coverage in the case of Agilent SureSelect 50Mb even if the latest has been sequenced using Fragment libraries on Solid3 system and the previous two using a Pair-end libraries on Solid4 system. This confirms that the shown slope is more dependent on the enrichment method than on the overall sequence produced (see also Figure S8). Examining the curve gene by gene (Figure S2) we can appreciate for example that in the specific case of USH2A, the Agilent SureSelect 50MB enrichment shows a worse performance compared with all the others.

### References

1. Applied Biosystems: BioScopeTM Software Users Guide.
2. http://picard.sourceforge.net/
3. Tang S, Hyland FCL, Wessel TC, Sorenson J, Peckham H, De La Vega FM. DiBayes: A SNP detection algorithm for Next-Generation dibase sequencing. Proceeding of the 12th Annual International Conference on Research in Computational Molecular Biology (RECOMB 2008). Singapore. March 30-April 2, 2008.
4. Wang K, Li M, Hakonarson H: ANNOVAR: functional annotation of genetic variants from high-throughput sequencing data. Nucleic Acids Research 2010, 38:e164.
5. Pruitt KD, Tatusova T, Klimke W, Maglott DR. NCBI Reference Sequences: current status, policy and new initiatives. Nucleic Acids Res. 2009 Jan;37(Database issue):D32-6.
6. Sherry ST, Ward MH, Kholodov M, Baker J, Phan L, Smigielski EM, Sirotkin K. dbSNP: the NCBI database of genetic variation. Nucleic Acids Res. 2001 Jan 1;29(1):308-11.
7. 1000 Genomes Project Consortium. A map of human genome variation from population-scale sequencing. Nature. 2010 Oct 28;467(7319):1061-73.
8. Kumar P, Henikoff S, Ng PC. 2009. Predicting the effects of coding non-synonymous variants on protein function using the sift algorithm. Nat Protoc 4 (7):1073–1081
9. Adzhubei IA, Schmidt S, Peshkin L, Ramensky VE, Gerasimova A, Bork P, Kondrashov AS, Sunyaev SR. 2010. A method and server for predicting damaging missense mutations. Nat Methods 7 (4):248–249
10. Chun S, Fay JC. 2009. Identification of deleterious mutations within three human genomes. Genome Res 19 (9):1553–1561
11. Schwarz JM, Rödelsperger C, Schuelke M, Seelow D. 2010. MutationTaster evaluates disease-causing potential of sequence alterations. Nat Methods 7 (8):575–576.
